# E-learning any time any place anywhere on mobile devices

**DOI:** 10.1007/s40037-013-0045-4

**Published:** 2013-03-30

**Authors:** Sylvia Eggermont, Peter M. Bloemendaal, Jary M. van Baalen

**Affiliations:** Leiden University Medical Center, Leiden, the Netherlands

**Keywords:** E-learning, New technology, Medical teaching, Mobile learning

## Abstract

The registered screen resolution of e-learning study moments in MedicalEducation.nl was used in this research to investigate the readiness of students and medical professionals to study e-learning on a mobile device. Between January 2008 and September 2012 the use of e-learning on a mobile device by students has quintupled to 2.29 %, while medical professionals lag behind in this development. If the use of mobile devices for e-learning is better supported, a rapid further increase should be anticipated. Further research on the desire of both students and medical professionals to study e-learning on a mobile device should be conducted.

## Introduction

Given the rise in the availability of Internet on mobile devices such as smartphones and touchpads and the advantage of e-learning as a just in time learning tool for medical students [1], it seems logical to develop medical e-learning Apps for mobile devices. Although students appreciate the availability of Apps for learning [2], the use of Apps compared with traditional Internet distributed e-learning has major drawbacks. Using Apps, valuable usage information on the learning material will be lost due to technical restrictions and the advantage of the close version control of traditional e-learning would be lost. Alternatively during development of e-learning programmes, one can adapt to the low resolution display of mobile devices and their specific screen touch function, enabling the use of traditional e-learning programmes on mobile devices and sidestepping the drawbacks of Apps.

This research was conducted to investigate the readiness of students and medical professionals to use traditional e-learning on mobile devices.

## The portal MedicalEducation.nl

**Fig. 1:**
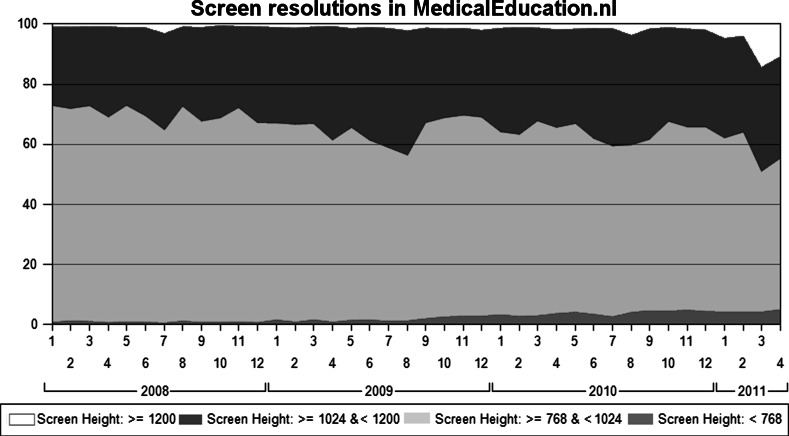
Distribution of e-learning sessions over the different screen resolutions in MedicalEducation.nl over time

MedicalEducation.nl http://medicaleducation.nl is used by all medical faculties in the Netherlands as the sole portal for the distribution of medical e-learning to students over the Internet [3]. MedicalEducation.nl provides access to modules that range from first-year medical school to postgraduate education in both level and subject and is therefore used by students as well as medical professionals [4]. The portal provides users with an overview of their past e-learning study activities and information on the additionally available e-learning modules. The main purpose of MedicalEducation.nl for users is to find, start and follow medical e-learning programmes for studying. Each start of an e-learning module by a user is registered as a session. Session information includes user, e-learning module, start time, duration and technical data such as the browser and user agent the learner uses. Session information provides teachers and course directors with feedback on the use of their study materials. The technical data provide e-learning developers with information on the conditions under which their e-learning is used. The display resolution has been extracted from this data since 1/1/2008 (Fig. 1).

## Question

- Is it allowed to conclude from the increased proportion of sessions with a small screen height in this figure that the use of mobile devices for studying e-learning has become established?
- If so, is the use of mobile devices for studying e-learning distributed equally over all user groups, or are there any exceptions?

## Method

As the main characteristic of mobile devices is the small display resolution, we extracted the use of mobile devices from the display resolution using a horizontal resolution cut-off at a maximum of 800 pixels and a vertical resolution cut-off at 768 pixels. All displays of desktop PCs and laptops fall out of this range and all smartphones and most touchpads fall within.

For each year the total numbers of individual users that have used e-learning on MedicalEdcuation.nl and the total number that have used it at least once on a mobile device were counted.

From all MedicalEducation.nl users, Leiden students and LUMC physicians were distinguished by their email addresses, as respective representative groups of students and medical professionals (see Table 1).

**Table 1:** Distribution of mobile device users for e-learning over representative student and physician groups

	All users			Leiden students			LUMC physicians		
	Total	Mobile	%Mobile	Total	Mobile	%Mobile	Total	Mobile	%Mobile
2008	10,943	24	0.22	2556	12	0.47	403	0	0.00
2009	14,796	105	0.71	2658	36	1.35	1710	3	0.18
2010	16,199	147	0.91	1913	54	1.85	876	1	0.11
2011	17,555	136	0.77	3015	36	1.19	1331	0	0.00
Sept-2012	13,679	195	1.43	2404	55	2.29	1016	4	0.39

## Results

The percentage of all users of MedicalEducation.nl that use a mobile device for e-learning has been gradually increasing since January 2008, but with 1.43 % in September 2012 is still considered low.

Among students, the use of mobile devices for e-learning has almost quintupled in the past 4 years and 8 months from 0.47 to 2.29 %. For the time being, medical professionals lag behind in this development.

## Discussion

Given the cut-off values for mobile usage, the percentage of users that have used e-learning on mobile devices is in reality higher than measured in this research. The technical session data could alternatively be used to distinguish mobile devices on their user agent instead of the screen resolution, but with so many different devices, this dataset would inevitably also remain incomplete.

For now, most traditional e-learning modules are not optimally designed for use on mobile devices, either because of a fixed screen resolution that does not fit the small display of the mobile device, or the use of interactions that are incompatible with the touch screen function. This might hold users back from using mobile devices for studying e-learning. If in the near future the use of e-learning on mobile devices is better supported, we anticipate a rapid further increase of usage of mobile devices by students for study purposes.

Inventory of the students’ and medical professional’s desire to study e-learning on a mobile device is advisable.
